# Oxygen desaturation following methylene blue injection: Not always spurious

**DOI:** 10.4103/1658-354X.76471

**Published:** 2011

**Authors:** Uma Hariharan, Rajesh Sood, Arindam Choudhury, Rakesh Garg, Jaswinder Kaur

**Affiliations:** *Department of Anaesthesiology and Intensive Care, Dr Ram Manohar Lohia Hospital, New Delhi, India*

Sir,

Methylene blue (MB) or tetramethylthionine chloride trihydrate or Swiss blue is extensively used in gynecology for diagnostic laparoscopies, apart from being used to identify cancerous lesions and lymph nodes.[1] MB, when injected intravenously or into the uterus or lymph node, can cause transient decrease in oxygen saturation (SpO_2_) without causing actual decrease in partial pressure of oxygen (PaO_2_) in blood.[2] We, hereby, report a case of oxygen desaturation combined with a concomitant fall in PaO_2_ in the patient’s arterial blood following MB.

A 30-year-old female weighing 50 kg was scheduled for diagnostic laparoscopy for assessing tubal patency following infertility. The patient denied history suggestive of any comorbidities except for infertility. In the operation room (OR), standard monitors were attached. General anesthesia was induced with intravenous fentanyl (100 μg), propofol (100 mg) and vecuronium (5 mg) and trachea intubated. Patient was positioned in lithotomy with 15° head-down tilt and pneumoperitoneum was created using carbon dioxide. Intraoperatively, 10 mL of dilute MB was instilled slowly into the uterine cavity by the obstetrician manipulating the vaginal end. After the tubes were visualized by blue staining, there was a fall in SpO_2_ from 100 to 88% over a period of 2 minutes. A sample for arterial blood gas analysis (ABG) was sent. Chest auscultation revealed equal air entry and occasional fine crepts bilaterally. The surgeons were asked to stop MB instillation and 100% oxygen was administered. The patient was made supine. SpO_2_ was increased to 92% and remained there for around 5 minutes. Tracheal tube suction revealed slight pink frothy solution. The ABG report (FiO_2_ 0.4) revealed pH 7.343, pCO_2_ 43.4 mmHg, pO_2_ 59.3 mm Hg, HCO_3_ 22.9 meq/L. SpO_2_ gradually improved to 98% over the next 10-12 minutes with positive pressure ventilation and on auscultation chest became clear. The residual neuromuscular blockade was reversed and trachea was extubated. In the recovery room, oxygen was given by face mask in prop-up position and the SpO_2_ was 99%. Repeat ABG revealed pH 7.387, pCO_2_ 35.7 mm Hg, pO_2_ 80.2 mm Hg, HCO_3_ 21 meq/L. Patient was shifted to the ward later and discharged on the third postoperative day uneventfully.

Pulse oximeter operates on the principle of Beer-Lambert law.[3] MB absorbs most of the 660 nm light emission and gives a false estimate of the percentage of oxyhemoglobin and SaO_2_[4] In large doses, MB causes methemoglobinemia by converting ferrous iron of reduced hemoglobin to ferric.

Initially, we thought that this desaturation was spurious secondary to interpretation of the dye as reduced hemoglobin by the spectrophotometer of the oximeter. But, ABG revealed decrease in PaO_2_ along with SaO_2_. Pulmonary edema following MB has been reported earlier.[5,6] In our case, the possibility of a mild form of pulmonary edema cannot be ruled out as there were thin watery secretions suctioned out of the tracheal tube. Presumably this was self-limiting. Our timely intervention of stopping further MB instillation, administering 100% oxygen by manual assist bag ventilation, tracheal tube suctioning and correction of patient position, resulted in an uneventful postoperative course. To conclude, patient showing oxygen desaturation following MB should not simply be interpreted only as being spurious, but should be investigated further by ABG analysis and if possible, by co-oximetry.
